# Evaluating the consistency of judgments derived through both *in silico* and expert application of the Cramer classification scheme

**DOI:** 10.1016/j.fct.2024.115070

**Published:** 2024-10-22

**Authors:** James W. Firman, Alan Boobis, Heli M. Hollnagel, Stefan Kaiser, David P. Lovell, Angelo Moretto, Severin Mueller, Cynthia V. Rider, Florian Schmidt, Szabina Stice, Sanjeeva J. Wijeyesakere, Geraldine Borja, Grace Patlewicz

**Affiliations:** a Liverpool John Moores University, Liverpool, United Kingdom; b Imperial College London, London, United Kingdom; c Dow Europe, Zurich, Switzerland; d DSM-Firmenich, Kaiseraugst, Switzerland; e St. George’s University of London, London, United Kingdom; f Università degli Studi di Padova, Padua, Italy; g Givaudan International SA, Kemptthal, Switzerland; h National Institute of Environmental Health Sciences, North Carolina, USA; i US Food and Drug Administration, Silver Spring, MD, USA; j Dow Inc., Midland, Michigan, USA; k International Life Sciences Institute Europe, Brussels, Belgium; l Center for Computational Toxicology & Exposure (CCTE), U.S. Environmental Protection Agency, Research Triangle Park, North Carolina, USA

**Keywords:** Cramer classification scheme, Toxtree, OECD QSAR toolbox, In silico, Risk assessment

## Abstract

The Cramer classification scheme has emerged as one of the most extensively-adopted predictive toxicology tools, owing in part to its employment for chemical categorisation within threshold of toxicological concern evaluation. The characteristics of several of its rules have contributed to inconsistencies with respect to degree of hazard attributed to common (particularly food-relevant) substances. This investigation examines these discrepancies, and their origins, raising awareness of such issues amongst users seeking to apply and/or adapt the rule-set.

A dataset of over 3000 compounds was assembled, each with Cramer class assignments issued by up to four groups of industry and academic experts. These were complemented by corresponding outputs from *in silico* implementations of the scheme present within Toxtree and OECD QSAR Toolbox software, including a working of a “Revised Cramer Decision Tree”. Consistency between judgments was assessed, revealing that although the extent of inter-expert agreement was very high (≥97%), general concordance between expert and *in silico* calls was more modest (~70%). In particular, 22 chemical groupings were identified to serve as prominent sources of disagreement, the origins of which could be attributed either to differences in subjective interpretation, to software coding anomalies, or to reforms introduced by authors of the revised rules.

## 1. Introduction

The Cramer classification scheme is a well-established framework enabling the categorisation of chemicals for purposes of hazard estimation (Cramer et al., 1978). It exists in the form of a decision tree, consisting of a series of 33 structure- and use-based questions (reproduced within Supporting Information 1), through which candidate molecules are ultimately assigned into one of three broad classes: “I” indicating substances of lowest concern, “III” highest and “II” intermediate. Since their 1978 development, these have each come to be associated with defined limits of daily oral exposure that can be presumed safe, thus placing them centrally within the application of threshold of toxicological concern (TTC) approaches to safety assessment (particularly in the context of food). (European Food Safety Authority, 2019; Serafimova et al., 2021).

It is acknowledged that several Cramer rules are formulated in a manner which sees their application open, in practice, to differing interpretations on the part of users (Patlewicz et al., 2008). In some instances, this appears by design: for example, the free and subjective definition of substances holding status as “Normal constituents of the body”, “Common terpenes” or “Common components of food” stands as a stated requirement. Naturally, the handling of such a task is dependent upon the reader’s knowledge and understanding with respect to physiology, organic chemistry and toxicology. Alternatively, there are occasions whereby unclear phrasing may render an apparently explicit, structure-based directive as ambiguous (Roberts et al., 2015). It is, therefore, inevitable that the adoption of the scheme for use across academic, industrial and regulatory settings has, over the course of four decades, led to emergence of non-aligning views concerning the placement of certain chemicals – coupled with efforts to impart revision and refinement to the conditions as written (particularly in light of advancements in scientific understanding) (Tluczkiewicz et al., 2011; Dewhurst et al., 2013; European Food Safety Authority and World Health Organization, 2016; Lapenna et al., 2011). Such variability will serve only to impart uncertainty concerning the extent of hazard which may be attributed to a substance – possibly culminating in the perceived need to conduct otherwise avoidable *in vivo* evaluation.

Aside from the potential for introduction of classification inconsistency, the manual application of the rule-set, particularly against larger compound collections, is liable to be a labour-intensive process. As such, *in silico* tools permitting the automated, bulk assignment of Cramer class have seen development (European Food Safety Authority, 2019). Amongst the most extensively-employed implementations are those present within Toxtree (https://toxtree.sourceforge.net) and in the OECD QSAR Toolbox (https://qsartoolbox.org) – both of which are freely-accessible programs (Patlewicz et al., 2008; Dimitrov et al., 2016). The original iteration of the scheme as integrated within Toxtree was devised with reference to datasets reported in the primary publication of Cramer et al., and later in Munro et al. (Cramer et al., 1978; Cramer rules, 2008; Munro et al., 1996). Concordance of assignments made by experts was evaluated by developers – as was the extent to which these might be reproduced by implementation of structural rules and logic.

As versatile as these resources undoubtedly are, the aforementioned ambiguous or open-ended nature of certain rules ensures that inherent challenges are associated with their realisation *in silico* (European Food Safety Authority, 2019). Accordingly, the classifications generated remain liable to differ from those derived courtesy of human judgment. In an effort to identify and articulate issues arising, researchers such as Lapenna and Worth, Bhatia et al. and Roberts et al. have performed analyses so as to compare and contrast the concordance between available expert and *in silico* assignments – highlighting those classes of chemicals most prone to be judged differently by each (Roberts et al., 2015; Lapenna et al., 2011; Bhatia et al., 2015). In doing so, authors were to note issues present within the rendering of 14 rules, in turn leading to mishandling of groups such as (amongst others) secondary and tertiary alcohols, alkyl ketones and cyclic esters. Origins of these anomalies were traced both to apparent errors in the interpretation and/or coding of defined structural requirements, and to insufficiencies within compound lists assembled for the purposes of addressing the more subjective queries.

Since its release, two additional, related tools have appeared upon the Toxtree platform. The first, “Cramer rules, with extensions” integrates a selection of five further queries, each with specialised remit, in and amongst those comprising the original series (European Food Safety Authority and World Health Organization, 2016; Lapenna et al., 2011; Cramer rules, 2009). It is with this that the sole iteration present within OECD QSAR Toolbox v.4.6, “Toxic Hazard classification by Cramer”, most closely aligns. The second, titled “Revised Cramer decision tree”, offers a more thorough reconstitution of the original approach – retaining essential features (general applicability and purpose, three-tier classification system etc.), whilst simultaneously adapting its judgment regarding the toxic potential associated with certain functional groups and, perhaps most crucially of all, removing all reliance upon user discretion within the evaluation of rule conditions (Revised Cramer Decision Tree, 2018; Schnabel et al., 2015; Rogers et al., 2020).

Documentation characterising the similarities and differences present between the tools within Toxtree is, in some instances, not comprehensive. Only its original Cramer implementation is associated with a peer-reviewed publication – whilst “Cramer with extensions” is instead supported by a detailed User Manual (Cramer et al., 1978; Cramer rules, 2009). However, no similar text relating to the “Revised Cramer scheme” may be traced. Accordingly, the scientific rationale underlying those amendments made during the course of its creation remains unclear. This in turn impacts upon the confidence which may be held in its conclusions. Since release in 2018, application has apparently been limited – with use being recorded in only a handful of publications (Rogers et al., 2020; Hua et al., 2022).

The intention of this study is to provide what is, to date, the most thorough evaluation with respect to the concordance and variation in Cramer class assignments emerging from alternative issuing sources. To achieve this, expert human judgments were gathered, culminating in the assembly of a data set consisting of greater than 3000 (predominantly food-associated) substances. Comparisons were made, as appropriate, between each of these verdicts and with those derived from Toxtree (both its implementation of the original scheme, and its extended and revised forms) and the OECD QSAR Toolbox. Having identified features of chemistry most liable to be associated with classification misalignment, an underlying rationale for each was sought. This centred upon assessment of discrepancies arising with respect to interpretation and implementation of individual rules. Alongside offering an expansion in scope over the studies of Lapenna and Worth, Bhatia et al. and Roberts et al., our work additionally presents what is, to our knowledge, the first in-depth analysis as to the operation of the revised decision tree (Roberts et al., 2015; Lapenna et al., 2011; Bhatia et al., 2015).

## 2. Materials and methods

### 2.1. Compilation of dataset

#### 2.1.1. Sourcing of expert-derived Cramer class assignments

Data were acquired from within three source collections – each consisting of series of substances for which expert-attributed Cramer classifications were present. The FEMA (Flavour and Extract Manufacturers Association) collection was provided by Dr Szabina Stice (US FDA). For each member compound, a class had been assigned by chemists enacting manual application of rules reported within Cramer et al. (1978). A second set, sourced by Dr Florian Schmidt (Givaudan), consisted of a list of substances relevant to IOFI (International Organisation of the Flavour Industry). Within this dataset, expert classifications originating from EFSA and JECFA (Joint FAO/WHO Expert Committee on Food Additives) were supplied, with the latter typically taken from the corresponding FEMA judgments. Whereas both inventories were composed of food-associated chemicals, predominantly flavourings and fortifying agents, a third collection, retrieved from Yang et al., had emphasis instead upon cosmetic ingredients (Yang et al., 2017).

Curation was performed upon each set, in order to ensure standardisation and consistency with respect both to substance identity and to representation of associated chemical structure. From their corresponding CAS registry numbers, DSSTox Substance Identifiers (DTXSID) for all entries were sought through use of the US EPA CompTox Chemicals Dashboard (comptox.epa.gov/dashboard; accessed 1st June 2023) (Williams et al., 2017). Accompanying SMILES strings were extracted, prior to their canonicalisation in OpenBabel software (v. 2.4.0; http://openbabel.org). (O Boyle, 2011) Substances for which a matching DTXSID could not be traced were excluded from further analysis, whereas those possessing ID but lacking a defined structure – such a mixtures and polymers – were retained exclusively for purposes of evaluating concordance in expert-derived classification. An overview of the composition of these sets, following the process of curation, is presented within Table 1. Only those labelled as holding “defined structure” were appropriate for application *in silico*.

#### 2.1.2. Creation of unified data inventory

Subsequently, these standardised collections were compared in order to assess the extent to which their membership overlapped (outcomes presented within Table 2). In total, 3337 unique substances were identified – of which 3255 possessed defined structure. A unified dataset was constructed from this list, whereby all associated expert judgments were retained. This is presented within Supporting Information 2.

### 2.2. Application of in silico Cramer classification tools

Three interpretations of the Cramer classification scheme, each present as automated decision trees within Toxtree software (v.3.1.0), were evaluated.

- Original Cramer rules, as implemented and described by Patlewicz et al. (Patlewicz et al., 2008; Cramer rules, 2008)
- Cramer rules, with extensions: based upon the above, albeit with inclusion of five additional rules addressing functional groups such as phosphates, benzene-like substances, divalent sulphur-containing compounds and unsaturated heteroatom moieties (European Food Safety Authority and World Health Organization, 2016; Cramer rules, 2009).
- Revised Cramer decision tree.

Whilst both original and extended forms each adhere very closely to the series of conditions laid out by Cramer et al., the revised tree instead deviates in several respects (Cramer et al., 1978; Revised Cramer Decision Tree, 2018). No documentation explaining the rationale underlying these alterations appears to have been released, and as such it is necessary to instead piece together information traceable both from the software itself and also from elsewhere. It can be ascertained that the tool appeared first within Toxtree’s v.3.1.0 (i.e., current at time of writing) release, that it was developed “for IOFI and FEMA”, and further that it “incorporates additional industry-derived data on metabolism, toxicity and biochemistry of compounds”. (Revised Cramer Decision Tree, 2018; Rogers et al., 2020).

The list of variations below has been assembled based upon user observation of its implemented form, and cannot claim to be exhaustive. Ostensibly, these include.

- Elimination of questions requiring reference to expert-assembled look-up lists of body constituents, common terpenes and common food components (i.e., R.1, R.16 and R.22 of standard scheme) (Schnabel et al., 2015).
- Compensatory restructuring and reinterpretation of rules relating to substances (such as simple heterocycles) commonly present within the above lists.
- Introduction of novel conditions, specific to the handling of (amongst others) α,β-unsaturated carbonyl-containing compounds, organohalides and organophosphates.
- Amendment to, or restructuring of, existing rules defining the classification of groups such as allyl alcohols, cyclic esters (i.e., lactones), ketones and thiols.
- Automated hydrolysis or reduction of molecules bearing susceptible functional groups, followed by screening not as parent substance but as post-breakdown products (i.e., the ester ethyl propanoate processed as distinct ethanol and propanoic acid units).

In addition to each of the above, a fourth implementation, present within the OECD QSAR Toolbox (v.4.6) under the title “Toxic hazard classification by Cramer”, was concurrently assessed. This rendering incorporates a configuration identical to that of the aforementioned “Cramer rules, with extensions” – whilst offering further, minor amendment to the classical forms of R.15, R.20, R.21, R.24, R.33. Documentation, provided alongside the software, details the nature of these alterations. For additional information concerning the arrangement of these schemes (both Toxtree and OECD QSAR Toolbox forms), including the wording of their various rules, please refer to Supporting Information 1. It should be noted that, within their earlier studies, Roberts et al. and Bhatia et al. each made use of Toxtree v.2.6.0 and OECD QSAR Toolbox v.3.1 (Roberts et al., 2015; Bhatia et al., 2015).

Through the above tools, Cramer classifications were sought for each of those 3255 compounds for which a discrete, characterised chemical structure was present. Whilst Toxtree was successful in processing all, the QSAR Toolbox was capable of generating output only for 3135.

### 2.3. Quantitative evaluation of Cramer classification concordance

Between each of the above sources, comparison was drawn with regards to assignments granted to shared substances. The extent to which such verdicts were seen either to agree or to disagree, as a collective, was quantified and described through application of two principal metrics – these being “Classification concordance” (i.e., general accuracy) and the Cohen κ statistic (McHugh, 2012). The latter was calculated in both weighted and unweighted forms, expressed alongside their lower and upper confidence intervals.

Further evaluation was performed with respect to correspondence in the handling of individual classes (i.e., I, II and III). *In silico* capacity to replicate expert judgment was, in each instance, expressed in terms of true positive rate (TPR), true negative rate (TNR) and positive predictivity value (PPV).When evaluating agreement either between pairs of expert panels or between *in silico* tools, intra-class concordance was instead determined (i.e., from all assignments of a particular class issued by the two sources, the proportion common to both).

### 2.4. Identification and characterisation of chemical groups classified with inconsistency

#### 2.4.1. ToxPrint enrichment analysis

ChemoTyper software (v.1.0; https://chemotyper.org) was adopted in order to generate ToxPrint molecular fingerprints (a total of 729) describing each of the 3255 eligible compounds (Yang et al., 2015). Once more, inter-source comparisons were subject to examination: shared substances were divided into seven pools – one corresponding to those whose classifications aligned, and the remaining six to each of the others, dependent upon the precise nature of their disagreement (e.g., with those judged to fall within Class I through the first source, but Class III in the second, forming one group). Through application of the χ^2^ test, frequencies of ToxPrint occurrence were compared between aligning and non-aligning molecules (pool-by-pool), in order that the identities of specific fragments overrepresented amongst each of the latter could be ascertained.

#### 2.4.2. Determining the origins of disagreement

Output from ToxPrint analysis was used for the purposes of directing the manual evaluation of those compounds which were subject to differing inter-source classification – thus ensuring that their defining structural features were characterised. In the interests of simplicity, a “unified” expert judgment was adopted for each chemical, with the exclusion of the small number of substances that had mismatching interexpert assignments. Similarly, the extended Toxtree scheme was dropped from consideration on account of the almost total alignment of its output with that of the original. As such, principal focus of comparison lay upon.

- Expert (unified) against each of the remaining *in silico* implementations
- Each *in silico* implementation against the other

A subsequent examination of scheme output readings allowed for the identification of those rules implicated, in each case, as the primary origins of disagreement.

## 3. Results and discussion

### 3.1. Dataset overview

Evaluation of unified dataset composition revealed that a considerable proportion of its 3337 member substances were subject to Cramer class assignment by two or more expert sources. Particularly notable was the degree of overlap present between the FEMA, EFSA and JECFA lists – with 1840 compounds, accounting respectively for 88%, 65% and 85% of each set, held in common. On account of its differing focus (cosmetic ingredients, as opposed to food-relevant chemicals), the Yang et al. collection possessed a profile that was, in many regards, distinct (Yang et al., 2017). Indeed, only 23.9%, 27.6% and 24.9% of its 543 members were noted to co-occur, respectively, within either the FEMA, EFSA and JECFA sets. Amongst the remaining ~400 entries were found selections of colourants (particularly azo dyes), antimicrobial or antioxidant preservatives (e.g., paraben and gallate esters) and insecticides (of the pyrethroid class).

**Fig. 1.**
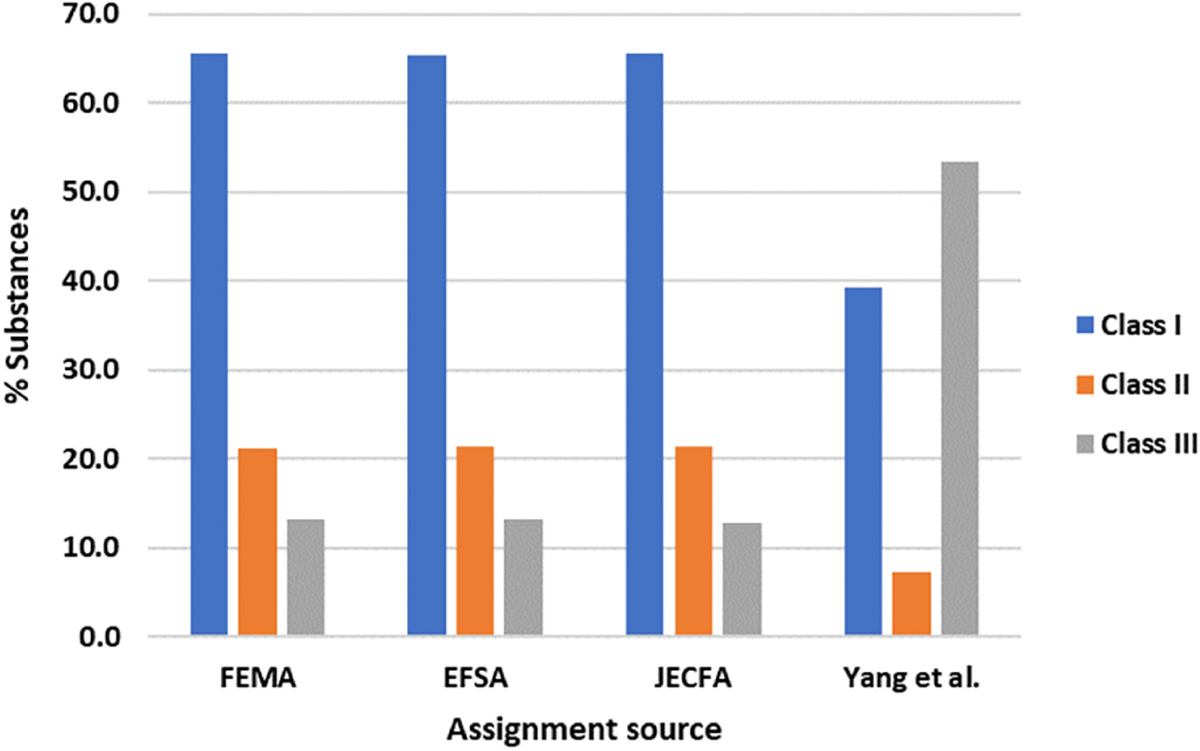
Distributions of expert-assigned Cramer classifications within each of the four primary data collections.

The general pattern of inter-set similarity and variation, as described above, was reflected within the distributions of class assignments present within each (Fig. 1). Whilst the FEMA, EFSA and JECFA collections were near-identical in this regard – with ~65% of their membership appearing as Class I, 20–25% as Class II and only 10–15% as Class III – Yang et al. was markedly different (Yang et al., 2017). Instead, an absolute majority of its substances found placement within Class III. This can again be considered reflective of the emphasis of each: contrasting comparatively inert food-associated substances against the more diverse range present within the latter.

### 3.2. Extent of concordance in Cramer class assignment

#### 3.2.1. Inter-expert judgment consistency

As is illustrated in Table 2 (unabridged confusion matrices presented Supporting Information 3), misalignment in classification issued by FEMA, EFSA and JECFA experts occurred only infrequently. In particular, it was noted that only 0.2% of corresponding FEMA and JECFA verdicts – representing four substances from out of 1969 shared – stood in disagreement with one another.

More substantial variation was, however, witnessed upon the comparison of each with the series of judgments presented by Yang et al. (82.7%–83.7% concordance, weighted κ of 0.67–0.68) (Yang et al., 2017). In order to rationalise this observation, it is important to consider the methodology which was adopted by the authors of that study when assigning classification. In essence, a hybrid approach was employed: class assignments were initially sourced either through the Munro et al. dataset or from Toxtree (v.2.6.13) – with readjustments made, as appropriate, in instances where expert curators felt that either of these verdicts may have been mistaken (Munro et al., 1996; Yang et al., 2017). It appears plausible that, at the heart of the increase in discordance, lay an inability to distinguish (and subsequently correct) certain erroneous classifications emerging from the software. This matter is further discussed within Section 3.3.2.

#### 3.2.2. In silico inter-scheme consistency

The passing of the collection of 3255 eligible substances through each of the four accessible *in silico* Cramer implementations allowed likewise for the consistency in their classifications to be evaluated. Selected results are presented within Table 3 (expanded in full under Supporting Information 4). Minimal variation was present between those outcomes acquired using the original and extended Toxtree forms – with only 12 compounds noted to differ in terms of their ultimate assignment. These belonged to a selection of sulphides (traditionally placed under Class I) identified within R.43 of the extended scheme as holding “potentially harmful divalent sulphur” – on account of which they are instead labelled Class III (European Food Safety Authority and World Health Organization, 2016). Agreement between outputs taken the original iteration, and those acquired courtesy of the QSAR Toolbox, sat at 82.6% (κ= 0.718). Insights into the origins of these misalignments – alongside all others – are provided within Section 3.3.

Discrepancy in judgment between Toxtree original and revised schemes occurred with much greater frequency – with general concordance standing at only 68.3%, and weighted κ at 0.525. This was to be anticipated: unlike the extended form, which was built atop the scaffold of the original, the revised tool was instead constructed seemingly from the ground upwards. Accordingly, it not only offers varying perspectives as to the potency of various specific chemical groups (e.g., allyl alcohol derivatives and α,β-unsaturated carbonyls), but further introduces subtle amendments in the implementations of several existing rules. As shall appear as a theme in subsequent analysis, it was in assignment of substances to Class II that relative extent of disagreement was most noteworthy (with only 5.71% of these occurrences proving common to both). For similar reasons, Toxtree revised and QSAR Toolbox verdicts were to accord in only 71.5% of instances (κ = 0.556).

#### 3.2.3. Expert judgment vs in silico output consistency

Assessment of the extent to which Toxtree and QSAR Toolbox scheme classifications accorded with those adopted by experts was deemed to be of particular importance. Illustrative outcomes of this analysis are presented in Table 4 – with the complete selection accessible as. Supporting Information 5.

Given the broad overlap present between the constitutions of the FEMA, EFSA and JECFA collections and collections, and the high degree of concordance in corresponding expert assignments, it is unsurprising that metrics related to their alignment with Toxtree and QSAR Toolbox output were, in each instance, very similar. Overall expert-*in silico* classification consistency sat between 69–72% (weighted κ 0.47–0.57), with no single implementation, despite variability with respect to one another, proving notably superior to its counterparts in this regard. However, closer inspection revealed divergence in the means by which individual classes were themselves liable to be handled. Both original and extended trees, alongside that within the QSAR Toolbox, displayed a shortfall in Class II assignments – with many such substances instead judged to lie within Class III. By contrast, the revised form sacrificed such conservativeness: Class II was to appear more frequently as a verdict, largely at the expense of Class III. This serves to reflect the tendency noted within 3.2.2.

The degree of alignment between Toxtree original/extended and Yang et al. calls was superior, standing at 87.2% in terms of concordance (and with weighted κ of 0.778). This is explicable with reference to points raised in 3.2.1, relating the role played by Toxtree within the class-assignment protocol as adopted by the study authors. Accounting for the extent to which the output of the revised scheme differed relative to that of the original implementation (as is discussed within Section 3.2.2), the observed reduction in judgment concordance (to 77.9%) was as anticipated.

### 3.3. Characterisation of the forms and origins of classification misalignment

#### 3.3.1. Expert vs Toxtree and Toxtree inter-scheme divergence

In examining the basis of classification inconsistency, it was first necessary to identify chemical features overrepresented across substances whose assignments were seen to clash. To guide this process, ToxPrint fragment key expressions were compared, in accordance with methodology described within Section 2.4.1. The subsequent exercise of manual evaluation (outlined Section 2.4.2) led to the definitive identification of 22 chemical families commonly serving as causes of intersource disagreement. Exemplified by groups including α,β-unsaturated carbonyls, simple heterocycle derivatives and polysulphides, their full listing may be found within Table 5. Deeper examination into the consistency of rule interpretation and implementation was performed, in order that the origins of these disparities could be rationalised.

In general terms, four sources of disagreement were identified.

- **Variation in opinion when applying rules requiring greater degree of subjective assessment:** These include R.1 (body constituents), R.5 (common carbohydrates), R.16 (common terpenes) and R.22 (food components) within the original scheme.
- **Misinterpretation with respect to wording of structure-based rules:**

Text of Cramer publication may, in certain instances, incorporate unintended ambiguity, potentially contributing factor towards apparent anomalies within expert judgments.

- **Essential differences between original and revised schemes:**

Opinion between scheme authors may not align with respect to degree of hazard associated with certain chemical groups (e.g., α,β-unsaturated carbonyls, allyl alcohols).

- **Apparent errors in coding, ensuring that a rule was not implemented as intended:**

Evident within both Toxtree and QSAR Toolbox schemes.

##### 3.3.1.1. Interpretation: rules requiring subjective evaluation. **Amino acid**

Unless explicitly identified under R.1 or R.22, amino acids will find assignment based upon the nature of their side-chain, reaching either Class I or (more commonly) Class III. It is R.1 which served as the source of much expert-Toxtree disagreement in this respect: whereas the latter regards each of the 20 standard, proteinogenic amino acids (in their L-configuration) as “normal constituents of the body”, the opinion of agency experts appeared to differ. Accordingly, it was common to see such compounds (e.g., L-cysteine, L-phenylalanine, L-arginine) classified “I” through Toxtree and “III” as consequence of expert judgment. By contrast, the experts had an ostensible preference for racemic, D,L-forms – with each seeing assignment to Class I, irrespective of structure.

The Toxtree revised scheme is, according to the text accompanying its R.2, intended to follow the lead of Toxtree’s original implementation – conferring Class I upon all L-amino acids. However, the rule is rendered ineffective by what would appear to be an error in coding. Not one of the compound varieties referenced within its description were, in practice, identified – (either L-amino acid, simple linear alkyl alcohol, carboxylic acid or aldehyde). Accordingly, all were passed on with a “No” judgment – albeit with a majority, by way of coincidence, proceeding onwards to an ultimate Class I verdict. Conversely, supplying the amino acid in its racemic form (e.g., D,L-Alanine) led to the apparently mistaken triggering of a “Yes” response.

Given that the wording to scheme R.1, as written by Cramer, reads “Is the substance a normal constituent of the body or an optical isomer of such?“, it seems that the drawing of distinctions between the configurations of the various acids is an act which runs counter to developer intentions (Cramer et al., 1978). However, experts are themselves noted to adopt this practice on occasion (assigning, for example, phenylalanine racemate to Class I and L-isomer to Class III). The QSAR Toolbox implementation does not account for configuration, and accordingly places all tested amino acids immediately within Class I.

###### Simple, substituted heterocycle

Amongst the substances most liable to experience inconsistency in judgement were those from series of substituted, monocyclic heterocycles typified by simple derivatives of furan, pyrazine and thiazoline. Cramer rules see many such compounds, courtesy of responses R.14 *N* (if aromatic) or R.12 *N* (if not), faced with verylikely assignment to Class III – unless they should then be identified, within R.22, as common components of food. It is at this point that divergence appears to occur, with agency experts being much more inclined to consider these families as holding the status of general dietary constituents. EFSA-published documents attesting the safety profiles of many may be retrieved – and it appears reasonable to assume that these compendia were used to inform judgments associated with the great majority here described (European Food Safety Authority, 2008a; European Food Safety Authority, 2017; European Food Safety Authority, 2021; European Food Safety Authority. Flavouring Group Evaluation 21, 2023). By contrast, Toxtree and the QSAR Toolbox adopt, for this purpose, very similar look-up tables. Amongst the issues associated with the lists employed (further explored within 3.4.2) are their small sizes: for example, through Toxtree, only five heterocyclic molecules (2-methylpyrazine, 2,5-dimethylpyrazine, nicotinic acid, maltol and ethyl maltol) were placed within Class II on direct account of membership. Unsurprisingly, these disparities can be said to contribute greatly towards misalignment in Class II-Class III (expert-Toxtree and expert-QSAR Toolbox) assignments, as was highlighted within Section 3.2.3.

It was necessary that the revised Cramer scheme make considerable changes to the handling of such substances, in order that it might classify them reliably in the absence either of direct expert input or of predefined lists. Amendments made to R.12 and R.14 led to the preferential placement of simple heterocycles (particularly non-aromatic) into Class II. Whilst clashes remained, with a number of such compounds nevertheless judged by experts as warranting a “No” response to original scheme R.22, general concordance was seen to increase.

###### Isothiocyanate

Again, many simple molecules bearing this functional group appeared to be identified by agency experts as common constituents of food – whilst concurrently being absent from the equivalent Toxtree and QSAR Toolbox lists (European Food Safety Authority, 2008b). Without intervention at R.22 (and hence Class II assignment), they proceed towards judgment as Class III. The revised scheme incorporates no specific reference to the group, with Class III likewise (by default) being its verdict.

###### Vicinal diketone

Owing to their possession of greater than a single ketone unit, all saturated alkyl 1,2-diones assessed under the original Cramer rule-set shall find themselves faced with R.22: the acyclic forms courtesy of answering “No” to R.20, and the cyclic through “No” at R.26. As such, it is most plausible that observed verdict discordance (experts assigning these substances to Class II, and both Toxtree and the QSAR Toolbox to Class III) was again a product of differences in opinion as regards their status as common food components (European Food Safety Authority, 2011). Placement within Class III was further noted to occur upon application of the revised scheme. Again, acyclic and cyclic paths converge (in this instance at its R.18). It is likely that the “Yes” response is triggered through matching of criteria described within 18(h), “vicinal diacetyl groups”.

###### Common terpene

The publication of Roberts et al. explores, in depth, the challenges associated with consistent application of R.16 and R.17 – namely, the definition of “common terpene”. These emerge from factors relating to the uncertainty surrounding the remit of the term “terpene”, the subjectivity inherent within the labelling “common”, and the practicalities of encoding the defining structural characteristics of such compounds *in silico* (Roberts et al., 2015).

Similar to the authors of that work, we noticed apparent disagreements between *in silico* and expert definition as relates to qualifying substances. Although it is not possible to state conclusively which of the examined terpenes owed their Class I expert assignment to R.16 response, it is the case that several likely candidates were overlooked by Toxtree (including linalool, nerolidol and various terpineol forms). Often tertiary alcohols, these generally found themselves judged as belonging to Class III, on account of an assumed error within the program’s rendering of R.20 (further considered within Section 3.3.1.3). Conversely, the sesquiterpene alcohol vetiverol was detected exclusively *in silico*. It should be noted that, whilst the methodology adopted by Toxtree in relation to the handling of R.1 and R.22 is made very transparent (i.e., application of openly-accessible look-up tables), the workings of its R.16 implementation remain unclear. Whether a corresponding list of terpenes is drawn upon, or whether structural rules are instead encoded, is not specified. The approach employed within the QSAR Toolbox is, however, viewable and rests upon the matching of general SMARTS-based (SMILES arbitrary target specification) conditions. Relative to Toxtree, resulting alignment with apparent expert evaluation is enhanced.

Within the revised scheme, R.6 assigns a Class I verdict to substances matching the description of “a monocyclic or bicyclic terpene (10 or 15 carbon terpene hydrocarbons)”. Restricted solely to hydrocarbons, its practical use is very limited. However, many of the screened terpenoids ultimately met with Class I judgment – and thus came to align with expert assessment – owing to their status as secondary or tertiary aliphatic alcohols. Unlike within the Toxtree interpretation of the original scheme, such functional groups were handled apparently without fault.

##### 3.3.1.2. Implementation: rules impacting upon broad substance range.

###### Salts

Implementation of Toxtree original scheme R.4 appears to be defective. Although intended to capture carboxylic acids, amines and sulphonic acids in common salt forms (thus ensuring that presence of the counter-ion does not see them assigned immediately into Class III), it was not successful in doing so. Substances matching the listed criteria, such as calcium lactate and sodium dodecyl sulphate, evaded detection. In contrast, organo-sulphonic acids presented without a counter-ion, such as taurine, mistakenly returned a “Yes” verdict. Although the revised scheme equivalent, R.5, was not universally ineffective (processing correctly the metallic salts of carboxylic acids and sulphonates), it nevertheless appeared incapable of recognising several of the groups supposedly within its remit. Amongst these were sulphate (organic or otherwise) and chloride/hydrochloride (inorganic). Similarly, the QSAR Toolbox form did display greater functionality – albeit with the caveat that on several occasions (e.g., in the instance of sodium diacetate), it was to issue a dual classification.

###### Complex aromatic

Cramer scheme R.30 is designed to separate out substituted benzenes in accordance with the nature of the functionalities attached: “complex” (those incorporating nitrogen or sulphur in any form, or alternatively carbon chains in excess of a certain length) or non-complex (shorter carbon chains, potentially bearing oxygen-based functional groups). Implementation of this question within Toxtree is dysfunctional – with each compound encountering it, irrespective of substituents borne, returning a “No” verdict (Roberts et al., 2015; Bhatia et al., 2015). Although it remains possible for substances answering “Yes” to receive an assignment to any class, the “No” outcome instead guarantees placement within Class I or II. As such, the consequence of this misapplication is that a variety of screened compounds find their hazard to be understated. Almost all of those evaluated as part of this exercise were granted a Class I verdict, clashing frequently with expert judgment. Amongst this number appear 1,4-benzenediamine, thiobenzene, benzyl octyl ether and enzacamene.

As with Toxtree, the QSAR Toolbox rendering of this rule does not identify nitrogen- or sulphur-bearing aromatics as “complex”. However, it possesses further issues distinct to itself. Contrary to stipulations within the Cramer et al. text, presence upon the ring of either a methoxy or hydroxy unit triggers a “No” response, irrespective of the nature of any further substitution (e.g., 2-mercaptoanisole). Conversely, although it is stated that account should be taken of the presence of “simple esters that may be hydrolysed to ring substituents of five or less carbons” (preceding “No”), such a requirement is not integrated appropriately. As such, whilst compounds such as 3-phenylpropyl hexanoate are seen to return a “Yes” verdict, ahead of ultimate Class II placement (clashing with expert judgment), the related methyl 4-phenylbutyrate is instead processed correctly. Within the revised scheme, equivalent conditions are distributed across its R.25, R.26 and R.27 – none of which were noted to display obvious dysfunction.

##### 3.3.1.3. Implementation of functional group: Alcohol.

###### Secondary alcohol

Roberts et al. and Bhatia et al. each highlight issues present within Toxtree’s implementation of Cramer R.18(b), which culminate in the mistaken returning of a “Yes” verdict (and an accompanying Class II assignment) for secondary alcohols (Roberts et al., 2015; Bhatia et al., 2015). The situation is perhaps a little more complex than it first appears: the influence of neighbouring substituents may still see that a compound bearing the unit is successful in avoiding the triggering of the alert (as in the examples of propylene glycol, 3,3,5-trimethylcyclohexan-1-ol and oct-3-en-2-ol). Amongst the substances classified incorrectly are found simple secondary alkyl alcohols (e.g., hexan-3-ol), alongside those bearing a combination of alkyl and aromatic substituents (e.g., 1-phenylpropan-1-ol). It should be noted that a variety of alcohols manage to bypass R.18, on account of their prior identification as common terpenes (as discussed within Section 3.3.1.1). No corresponding issues are apparent within either the QSAR Toolbox and Toxtree revised schemes, with expert classification commonly matched through each.

###### Tertiary alcohol

The Cramer rules makes no specific reference to the tertiary alcohol group. Simple substances bearing the unit would be anticipated to receive judgment through R.18, with the outcome being placement either into Class I (most likely) or Class II. A variety of terpene alcohols (such as linalool and cubebol) are tertiary in form, and as such may see default Class I allocation as a consequence of positive response to R.16. In alignment with these interpretations, expert evaluation does indeed lead to the predominance of a Class I verdict, whether terpene or otherwise (e.g., 2,3,4-trimethyl-3-pentanol, 2-methyl-1-phenylpropan-2-ol). However, some uncertainty remains with respect to the rationale which might, in some instances, have driven assignment. For example, expert Class II judgments granted to 2-methylbutan-2-ol and 2-methylpentan-2-ol are not immediately explicable based upon our reading of the rules. In this regard, experiences align with those of other authors (Roberts et al., 2015; Bhatia et al., 2015).

The Toxtree rendering of the scheme mishandles this group. In the great majority of instances, this leads to a Class III placement. Roberts et al. were able to identify those questions specifically responsible for incorrect routing: R.20 (inappropriate “No”) in open-chained forms, R.24 (inappropriate “No”) in alicyclic. Only aromatic molecules, through evading both, are capable of reaching R.18 (and allocation to Class I). Such anomalies again appear absent from both revised and QSAR Toolbox variants.

##### 3.3.1.4. Implementation of functional group: Carbonyl.

###### α,β-Unsaturated carbonyl

Only a limited selection of α,β-unsaturated carbonyl compounds are distinguished through the original scheme – each under the remit of R.18. Amongst this number (allocated to Class II where they would otherwise be assigned Class I) appear acrylic and methacrylic acid, acrolein/methacrolein, and substances bearing a ketone group situated adjacent to a terminal vinyl unit. Available evidence suggests that such conditions are implemented both in Toxtree and in the QSAR Toolbox without issue, and thus do not to contribute to misalignment in any form.

However, the revised scheme expands upon this list, further considering any compound bearing “An α,β-unsaturated aldehyde or ketone with no or one β-carbon substituent” to be worthy of Class II assignment (R.18). Cinnamaldehydes and rose ketones (such as the ionones and damascenones), which would otherwise fall within Class I, are amongst the families of substances impacted by the amendment.

###### Ketone (other aliphatic)

Open-chained, aliphatic mono-ketones were a further class to be granted attention through Roberts et al. and Bhatia et al., who identified inconsistent interpretation of R.18(h) as the origin of judgment discordance (Roberts et al., 2015; Bhatia et al., 2015). According to their hypothesis, unclear wording of the rule text is liable to serve as a source of confusion for scheme users, who may be uncertain as to whether one – or alternatively both – sides of the carbonyl unit should require chain lengths of four or greater carbon atoms in order for Class II to be triggered. Whereas the authors of those works favoured the latter reading, both agency experts and Toxtree opt generally for the former.

**Fig. 2.**
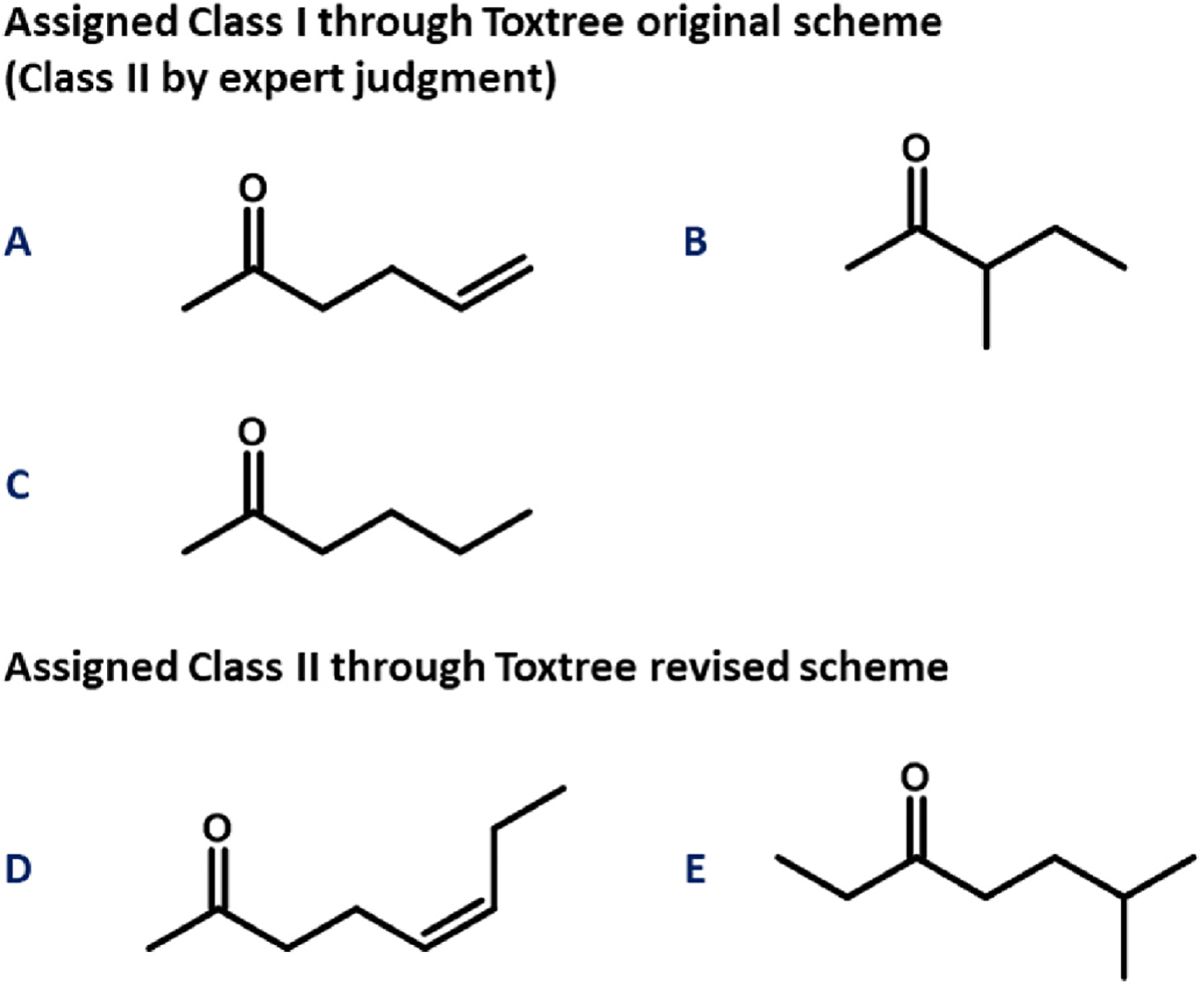
Selection of ketones representative of forms apparently mishandled within Toxtree. Through the application of the original scheme, Class II assignment would be anticipated for substances A (hex-5-en-2-one), B (3-methylpentan-2-one) and C (hexan-2-one). Conversely, substances D (*cis*-5-octen-2-one) and E (6-methylheptan-3-one) are characteristic of those which, according to revised scheme wording, ought to be placed into Class I.

However, anomalies remained present. Toxtree placed into Class I, contrary to expert assessment, several ketones which nevertheless bore four-or-more carbon atoms upon a single carbonyl wing – including Fig. 2, substances A (incorporating a distant alkene unit) and B (branched-chain). At the same time, Fig. 2, substance C (the saturated equivalent of the former) was assigned by the program as belonging to Class II. Given their lack of apparent alignment with the rules as written, these outcomes would suggest presence of coding errors.

Aside from the identification of substances suitable for definition either as body or general food constituents, or as terpenes, scheme implementation further requires discretion with respect to characterisation of “common carbohydrates” (R.5). Whilst it is not possible to identify major issues with the functioning of this rule as rendered within the Toxtree original or revised forms (R.6 within the latter), its QSAR Toolbox working (as R.5-B) does display an apparent shortcoming. This sees the erroneous labelling of all saturated, unsubstituted alkyl ketones as carbohydrates (resulting in their immediate Class I assignment). It should be noted that unsaturated variants instead proceed through to R.18. However, the classifications granted to this set ultimately match the forms generated within Toxtree, as opposed to those bestowed by experts, thus suggesting the presence of related concerns.

Through the revised scheme, linear alkyl ketones were placed within Class I – irrespective of carbon chain length. Since no mention of such restrictions is provided within the text accompanying its R.18, it is assumed that this is the intention of the developers. Variants incorporating, at the carbonyl γ-carbon, either an olefinic unit or a form of alkyl chain-branching (Fig. 2, substances D and E respectively) instead answered “No” at R.16, and were thus sent towards a Class III verdict. The reasoning behind this remains unclear, and it would appear to be unintended.

##### 3.3.1.5. Implementation of functional group: Sulphur-containing.

###### Thiol and thioether

There is much inconsistency with regards to the classifications granted to substances bearing sulphur-containing functional groups. Within the original Cramer scheme, it is R.20 which is typically responsible for deciding the fate of such compounds (unless cyclic). Its text states that, in order to return a “Yes” verdict, molecules should possess no greater than “one each of one or more” from a list of features, including thiol (i.e., mercaptan), thioether (i.e., mono-sulphide) and polysulphide. The corresponding conditions within the revised series are found within its R.16. Whilst these consider thioethers in a manner which is identical to the original, they are stricter with respect to thiols and polysulphides – both of which require the presence of alternative functionalities upon the profiled substance if “Yes” is to be triggered.

**Fig. 3.**
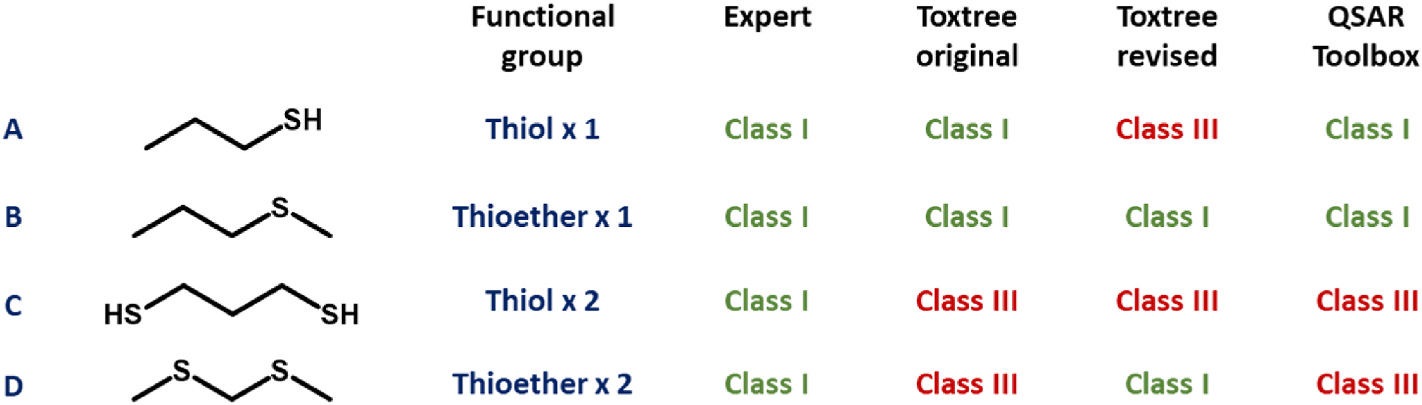
Variation in expert- and *in silico*-assigned Cramer classifications, applicable to acyclic alkyl mono-thiols (substance A: 1-propane-1-thiol), mono-thioethers (substance B: methyl propyl sulphide), di-thiols (substance C: propane-1,3-dithiol) and di-thioethers (substance D: 2,4-dithiapentane).

Toxtree’s interpretation of the original scheme, alongside that of the QSAR Toolbox, returned what appeared to be correct judgments when processing (through R.20) acyclic thiols and thioethers. Should either of these groups have been present only once, then compounds (exemplified, respectively, by Fig. 3 substances A and B) were sent towards Class I assignment. If greater than once, then Class III was highly likely. Agency experts, by contrast, placed both di-thiols (e.g., Fig. 3, substance C) and di-thioethers (e.g., Fig. 3, substance D) into Class I. This would look to be in contradiction to the wording of R.20. Meanwhile, the revised scheme assigned all corresponding compounds containing solely thiol functionalities to Class III, and those containing solely thioether to Class I (irrespective of number present). In the instance of thioethers, the wording and implementation of its R.16 appeared not to align.

###### Polysulphide

**Fig. 4.**
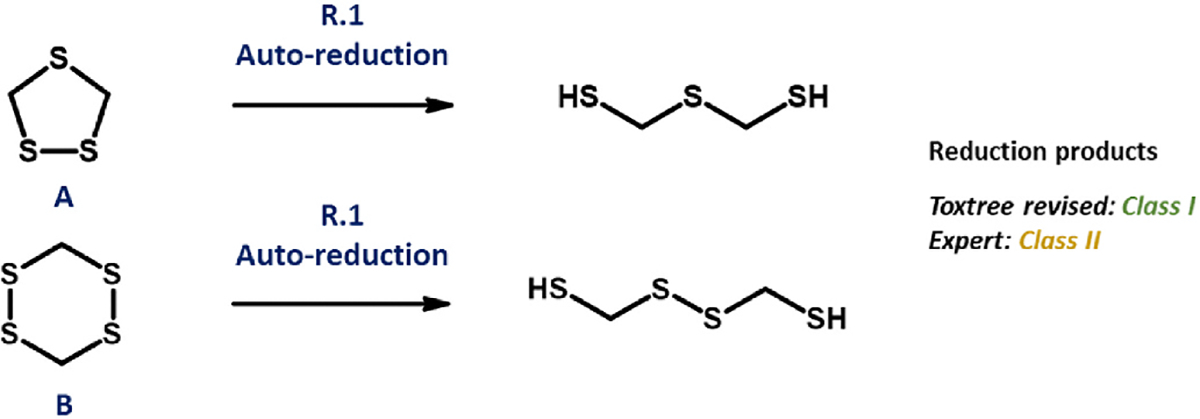
Scheme outlining origin of discrepancy present between expert- and Toxtree revised scheme-assigned Cramer classifications, as relates to selected cyclic polysulphides. Substance A: 1,2,4-trithiolane, substance B: 3,6-Dimethyl-1,2,4,5-tetrathiane.

With regard to acyclic, aliphatic polysulphides, both Toxtree schemes return a Class III verdict – in opposition to Class I, as granted by experts and through the QSAR Toolbox. In the instance of the original, this appears to emerge as a consequence of a coding error within R.20. Although the revised form (in its R.16) contains text relating to the disulphide unit, the feature is in fact subject to reduction at R.1, thus yielding a thiol pair. An apparent error within coding of revised R.16 is responsible for the placement into Class I of a specific series of heterocycles (exemplified by Fig. 4, substances A and B). These are five- or six-membered alicyclic compounds bearing a minimum of three ring sulphur atoms, of which at least two must be present as a polysulphide. Reduction, in each instance, yields products possessing a minimum two thiol groups – which ought, in accordance with wording of 16(c), to elicit a “No” response. However, a “Yes” outcome, leading ultimately to Class I assignment, instead results. The reasoning behind this is uncertain, but it is almost certainly associated with the co-occurrence of either thioether or polysulphide. Whereas expert judgment favours Class II, Toxtree (original) instead sees these substances allocated to Class III. Once more, R.22 is seemingly the most rational explanation for such variation.

##### 3.3.1.6. Implementation of functional group: Cyclic ester.

###### Lactones

**Fig. 5.**
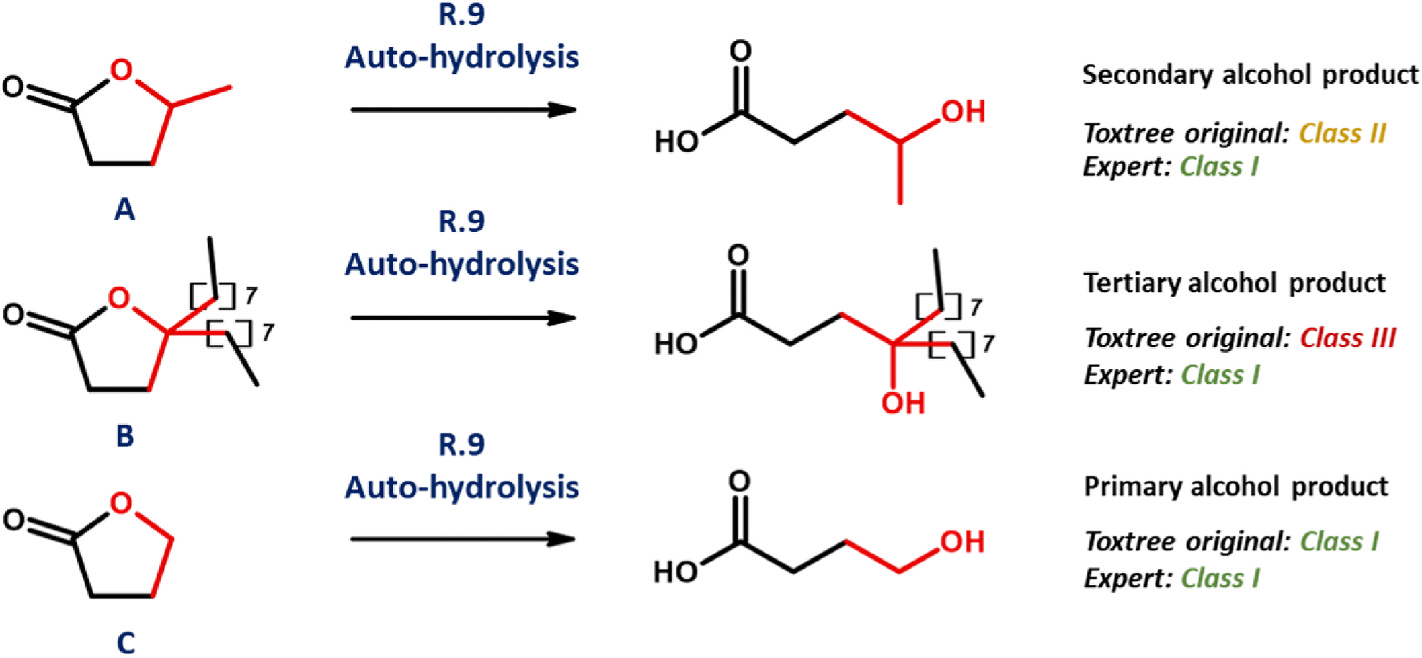
Scheme outlining origin of discrepancy between expert- and Toxtree-assigned (original scheme) Cramer classifications, as relates to unsaturated, unfused lactones. Substance A: pentano-1,4-lactone, B: 4-butyloctano-1,4-lactone, C: butyro-1,4-lactone.

It is R.9 which is integral in determining the fate of lactones. Within the original scheme, α,β-unsaturated or ring-fused equivalents are assigned immediately, upon answering “Yes”, to Class III – whereas all others are sent, in their hydrolysed form, to R.20. From this point, placement into each of the three classes is possible: albeit with the substances dominant amongst this particular dataset tending, by the wording of the rules, towards Class I. Toxtree implementation of R.9 is seemingly accurate, with almost all of those compounds registering a “Yes” response likewise seeing allocation by experts to Class III. However, with respect to those eliciting “No”, our observations are identical to those noted by Roberts et al. – whereby hydrolysis products, as alcohols, are subject to the very same issues in interpretation as are described within Section 3.3.1.3 (Roberts et al., 2015). Accordingly, saturated, branched lactones such as Fig. 5, substance A (with its secondary alcohol product) and 4-butyloctano-1,4-lactone (Fig. 5, substance B, with its tertiary alcohol product) are placed, respectively, into Class II and Class III – clashing with the verdicts of experts, who judge all as belonging within Class I. Only in instances where branching is absent at the carbonyl-adjacent carbon, such as Fig. 5, substance C (with primary alcohol product) does Toxtree return a Class I assignment. Such issues are absent within the QSAR Toolbox form.

In contrast, the revised form of the scheme generally views lactones as holding reduced toxic potential. Its R.9 incorporates a higher degree of resolution than does the original form: rather than allocating all α,β-unsaturated or ring-fused varieties to Class III, it is only phthalides and coumarins (of those screened) which are automatically granted such status. All others are judged in their hydrolysed form, resulting almost exclusively in Class I placement (secondary and tertiary alcohols being handled without error).

##### 3.3.1.7. Implementation of functional group: Acyclic ester.

###### Allyl alcohol ester

Toxtree and expert judgment accord almost perfectly with regards to the classification of allyl alcohol esters. Whilst QSAR Toolbox assignments likewise align with respect to cycle-containing variants, the purely open-chained are instead placed into Class III (as a consequence of R.44). The revised scheme, however, perceives all such substances to be of greater concern: whereas R.18 within the original form sees “allyl alcohol or its acetal, ketal or ester derivative” placed within Class II, its revised equivalent states that “allyl alcohol or its ester” instead warrants a Class III verdict.

###### Anthranilate ester

Amongst all highlighted groups, the anthranilate esters constitute a unique case: whilst corresponding expert and Toxtree original verdicts are in agreement, it is apparent that at least one of these sources is mistaken. Toxtree’s handling was almost certainly incorrect, placing the substances into Class I as a consequence of previously-attested difficulties relating to operation of R.30 (Section 3.3.1.2). However, short of returning a “Yes” response at R.1, it is unclear as to why expert evaluation should result in identical assignment. Although anthranilic acid is known to feature within metabolic pathways active in humans, its ester derivatives are not. By contrast, their presence in food is well attested (European Food Safety Authority, 2008a). Likewise, aforementioned R.30 insufficiencies are seen to impact upon the handling of these compounds through the QSAR Toolbox: again, at the root of this lies a failure to correctly flag nitrogen-containing aromatics. Those which reach Class III do so as a consequence of invoking a prior “Yes” verdict at R.2. This is a potentially dubious outcome, given that the Cramer et al. text speaks only of “aliphatic secondary amines”. Alongside other such “complex aromatics” (as described within 3.3.1.2), a Toxtree revised scheme Class III verdict was to arise as consequence of passage through R.25, R.26 and R.27.

##### 3.3.1.8. Implementation of functional group: Other.

###### 1,3-Dioxolane

**Fig. 6.**
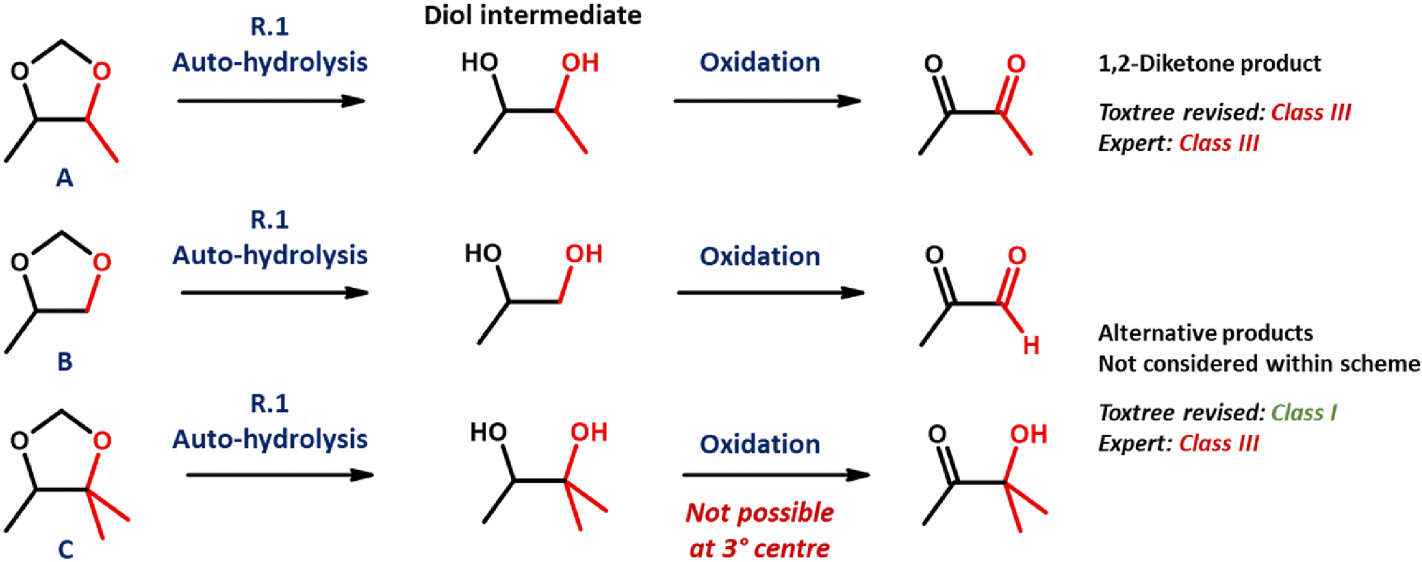
Scheme outlining origin of discrepancy between expert- and Toxtree-assigned (revised scheme) Cramer classifications, as relates to 1,3-dioxolanes. Substance A: representative 4,5-disubstituted derivative B: representative 4-substituted derivative, C: representative 4,5,5-trisubstituted derivative.

The 1,3-dioxolane unit is recognised directly as heterocyclic (R.7) within both Toxtree (original) and QSAR Toolbox scheme implementations, after which its general characteristics ensure that the returning of a Class III verdict is all but guaranteed (aligning with the call of experts). However, handling within the revised equivalent is very different. Automated hydrolysis of the group, upon passing through R.1, leads to its subsequent consideration in the form of a 1,2-diol product – the branching of which dictates the nature of the response elicited at R.18 (please refer to Fig. 6). Should both of these alcohol functionalities be secondary (as within the product emerging from Fig. 6, substance A), then “Yes” is triggered – seemingly through matching of 18(h) – resulting in assignment to Class III. However, in the great majority of instances, this will not be the case. Should the diol incorporate at least a single primary or tertiary alcohol, and thus hold invulnerability towards oxidation to a vicinal dione form (see also Section 3.3.1.3), then placement within Class I shall instead be the result.

###### Ether

There are two scenarios within which the presence of an ether linkage might lead to assignment of a substance to Class I courtesy of Toxtree’s working of the original scheme, both of which were explored by Roberts et al. (2015) The first concerns the mistaken issuing of a “Yes” response to R.20, and is relevant to a majority of open-chained compounds bearing the unit (dependent upon its position). As a consequence, these are spared from a near-guaranteed placement within either Class II (if identified as food component) or Class III. The second applies to alkoxy-substituted benzenes, and arises owing again to the general fault in R.30 (Section 3.3.1.2). Issues with the corresponding QSAR Toolbox rules may be seen to produce very similar effects.

Within the revised Cramer scheme, it is R.16 which is responsible for much of the function served, in the original, by R.20. Rather than overlooking the group, R.16 gives it consideration, permitting methoxy and ethoxy units (within open-chain molecules) to trigger “Yes”, provided that all accompanying conditions are met. Unless proceeding to match with features described in R.17 and R.18, then Class I assignment shall be the outcome (e.g., as in prenyl ethyl ether). With issues associated with the detection of complex aromatic substituents now absent, substances such as benzyl methyl ether and benzyl octyl ether are able to avoid a Class I verdict.

#### 3.3.2. Inter-expert divergence

As is outlined within Section 3.2.1, clashes between corresponding FEMA, EFSA and JECFA assignments were uncommon. Accordingly, ToxPrint expression analysis (as informs the findings discussed within Section 3.3.1) was, in this instance, deemed impractical and not informative. Rather, direct manual examination of features characterising inconsistently-handled substances was performed.

It was variation in the judgment of heterocyclic substances which was, as with Toxtree, to serve as a prominent source of any disagreement. For example, a sum of 21 furan derivatives were placed within Class II by both FEMA and JECFA, but instead Class III by EFSA (of the total 60 FEMA-EFSA and 67 EFSA-JECFA misalignments). These are joined by, amongst others, a further scattering of a thiazoles and thiazines. Once again, it is highly likely that, behind such discrepancy, lies a difference in opinion with respect to the status of these compounds as common food components. Should a formal verdict as to the general safety of such groups have been unavailable to a particular body of experts at the time of issuing their judgments, then Class III would have been the appropriate destination. In a similar manner, it is possible that lack of consensus concerning the identities of “common” terpenes (or their derivatives) may account for divergence in the classifications of (l)-α-bisabolol and isobornyl 2-methylbutyrate. These examples aside, the origins of inter-expert disagreement were generally opaque. Minor clusters, such as α-pentylcinnamyl derivatives, lactones and an assortment of dioxanes and dioxolanes, were present amongst the substances remaining. However, without knowledge of underlying rationale, it is challenging to locate precisely the points within the scheme at which judgment may have split.

Yang et al. assignments deviated from those of FEMA and JECFA on 22 occasions, and from EFSA on 26 (i.e., each of the aforementioned 22, with a further four exclusive). Section 3.2.1 relates how Toxtree output played a central role within the process by which Yang et al. reached their conclusions on class placement (Yang et al., 2017). As such, it is unsurprising that, from the 25 of those 26 substances which possessed defined structure, 19 (i.e., 76%) had a classification matching that acquired from the Toxtree original scheme. Amongst these were examples of putative body constituents (amino acids), food components (e.g., heterocycles and vicinal diketones) and also common terpenes. The remaining six, manually adjusted by the study authors, included caffeine, glutamine and L-cystine.

### 3.4. Mitigation of inconsistency in application of classical Cramer scheme

#### 3.4.1. Execution of explicit, structure-based rules

Issues within the implementation of explicit structural requirements were traced as the origin of disagreement in ten from the 16 compound groupings for which expert and Toxtree original scheme verdicts ultimately misaligned. On eight such occasions, this outcome is almost certain to have arisen as a function of errors on the part of software handling with respect to key substance features – either through the misinterpretation of rule text, or through the inappropriate coding of the characteristic molecular fragments. R.4 and R.30, for example, appear almost entirely inoperative, whereas R.18, R.20 and R.24 are functional only in part. To such a list may be added those which were further identified by other authors, yet nevertheless not associated with significant issues amongst the collection of compounds here examined: R.11, R.26, R.29 and R.32 (Roberts et al., 2015; Bhatia et al., 2015). QSAR Toolbox workings of R.4, R.18 and R.20 and R.30, whilst possessing greater general operability than their Toxtree counterparts, are nevertheless still not without issue.

In each instance, it is highly likely that these represent “bugs” within the software, which thus can be rectified. This aside, it has been acknowledged that the definition of structural properties including “simply branched” and “sterically hindered”, as appear within the primary Cramer et al. text, may benefit from clarification (Cramer et al., 1978; Roberts et al., 2015). Within three cases, the judgment of experts may instead have been founded upon misapplication. Each of these scenarios – poly-thiols and poly-thioethers (as detailed within Section 3.3.1.5) and the tertiary alcohol minority (Section 3.3.1.3) – can be plausibly related to R.18. Whilst the handling of anthranilate esters (Section 3.3.1.6) potentially represents a fourth example of this phenomenon, coincident with Toxtree R.30 error, it was nevertheless not possible to assert this claim definitively. Such anomalies may have arisen owing to conscious decision, on the part of experts, to apply personal knowledge outside of the strict rule framework.

#### 3.4.2. Approaches for the management of subjectivity

As previously discussed, the subjectivity inherent within the application of R.1, R.15, R.16, R.17 and R.22 represents a particularly challenging obstacle for those seeking recreation of the scheme in an automated form. In relation to R.1 and R.22, Toxtree and QSAR Toolbox developers approach the issue rationally – encoding within their software a pair defined substance lists: the first consisting of approximately 400 compounds identified as “normal constituents of the body”, and the second of little over 100 “common components of food” (Lapenna et al., 2011). The adequacy of the former is difficult to judge, since not only is the rationale underlying the inclusion and exclusion of substances not reported, but it further remains unclear as to precisely which expert Class I judgments arose definitively as a consequence of R.1 “Yes” response. It is, however, the case that 51 compounds, from the Toxtree 403, were present amongst the wider dataset (each may be found identified within Supporting Information 2). Following the discounting of Yang et al. exclusives, owing to their influence from Toxtree (Section 3.3.2), this number was to fall to 44. Of these, 29 were judged by experts to lie also within Class I, with thirteen within Class III and two within Class II. Amino acids were to constitute twelve from the fifteen misalignments. As discussed within Section 3.3.1.1, a removal of specifications relating to their stereochemistry would help ensure greater consistency, whilst further aligning with apparent instruction outlined within the Cramer et al. text (Cramer et al., 1978; Lapenna et al., 2011).

It is clear, however, that R.22 is an area of greater concern. As is referenced again under Section 3.3.1.1, both Toxtree and the QSAR Toolbox judge as “common components of food” only a fraction of the substances considered as such by experts. Toxtree’s reference table of 104 compounds is, in itself, seemingly compact. However, further analysis was to reveal that, in practical terms, this number is smaller still – with only 23 ever reaching the R.22 node (Lapenna and Worth were to report that 16 did likewise within Toxtree v.1.6) (Lapenna et al., 2011). Of the remaining 81, seven were found within the corresponding “body constituents” list and 74 were instead funnelled through alternative tree pathways (48 of the latter meeting eventually with a Class I assignment). Nine of the 23 were found to bear an expert classification. Following exclusion of two covered only through Yang et al., six of this remaining seven were likewise to fall within Class II (please refer to Supporting Information 6 for additional detail). If opinions are to align in these regards, then it is apparent that the present Toxtree table must be overhauled. Future releases of the software may feasibly integrate expanded forms of such a list, taking into account current consensus regarding the commonality and safety status of food ingredients (e.g., as expressed within EFSA guidance) (Dusemund et al., 2012). Alternatively, such an inventory might be maintained as a distinct and evolving document, incorporating references and rationales for each substance, accessible to users so that they might integrate it within the scheme courtesy of the program’s editing function. As is described within Section 3.3.1.1, the mechanism which underlies Toxtree implementation of R.16 is difficult to discern – with the task of identifying all “common terpenes” perhaps, in any case, now unrealistic (Roberts et al., 2015).

In summary, the use of poorly-defined criteria, such as the common occurrence of a compound either in the body or in food – coupled with the adoption of related look-up lists which lack transparency in relation to their composition – represents a scientifically-questionable approach which further serves as a ready source for judgment discrepancy. Some substances occurring as normal body constituents, or as food components, may nevertheless act as toxicants. The scientific community, together with regulatory stakeholders, will have to reach consensus regarding a point in time at which this more pragmatic grouping approach might be set aside (if it is deemed desirable to do so).

### 3.5. Scheme revision and evolution

It is beyond the scope of this study to cast judgment upon the validity of the rules expressed within the various iterations of the Cramer scheme, or else to suggest areas in which they might (further) be amended. Insightful discussion on this matter may instead be found elsewhere (Tluczkiewicz et al., 2011; Dewhurst et al., 2013; European Food Safety Authority and World Health Organization, 2016; Lapenna et al., 2011). Accordingly, the changes adopted within the revised form, as regards the extent of toxicity which may be associated with certain substance groups (e.g., α,β-unsaturated carbonyls, allyl alcohols), shall not be commented on. Published guidance relating to the conditions would, in any case, be essential in order for this to feasible.

However, on account of their unique circumstances, developer efforts to reassess the evaluation of heterocyclic substances can be addressed. Evidently, these alterations were introduced in light of the removal of list-based questions, as a means of rectifying the tendency of the original scheme to place these compounds within Class III. However, their uniform placement within Class II may further represent an oversimplification, leading instead to an underestimation of toxic potential. It is likely that only a re-analysis of available source data might allow for the drawing of appropriate structure-activity relationships, thus permitting the derivation of refined rules accounting for the handling of more specific sub-classes. As within the original scheme, rule functioning was liable to be impaired on account of errors in coding. Defects within revised R.2, R.5, and R.16 were detected, which impacted upon the processing of groups not limited to amino acids, salts, polysulphides and selected ketones. This list cannot claim to be exhaustive. In addition, the consequence of R.1 hydrolysis/reduction upon the ability of substances to reach the rules intended for their handling (as was witnessed with regard to polysulphides) is a matter requiring further examination.

## 4. Conclusion

Comparison of class assignments emerging from a total of up to eight sources, across a dataset consisting of greater than 3000 substances, has enabled comprehensive examination into both the frequency and origin of inconsistencies liable to arise within application of the Cramer classification scheme. Issues within the processing of 22 chemical groups were identified, with additional caution advised in the evaluation of compounds bearing these functionalities.

It wasnoted that, although expert judgments tended overwhelmingly to align with one another, the degree of concordance between these and *in silico* tool output was less impressive. In several instances, this variance was readily attributable to direct software shortcomings – often arising as a product of apparent coding anomalies with respect to the recognition of specific structural forms. Although this very obviously represents a problem, given the popularity of the platforms, it is anticipated that it should be readily addressable within its future releases. Where applicable, it was subjective definition (primarily a function of the limited scope of the Toxtree/QSAR Toolbox lists of “look-up” substances) which was, for the most part, otherwise responsible. Occasions were to arise, however, in which expert verdicts were themselves not explicable based upon our interpretation of rule text.

In future developments concerning chemical grouping as applied to TTC, it appears desirable to rely exclusively upon structural criteria. However, this would require a more complete knowledge of relevant structure-activity relationships than that which would have been available at the time of the initial Cramer publication. As such, our analysis was extended so that it encompassed performance of the 2018 “Revised Cramer Decision Tree” – a tool which introduced a variety of amendments to the original 1978 series, each with the apparent intention of removing elements of subjectivity. Whilst Issues with respect to implementation of certain conditions were identified, a lack of existing documentation ensured that the general merits of the scheme (in isolation) were challenging to assess. Numerous insights into its form and operation were, nevertheless, able to be acquired.

Both the original Cramer et al. publication, as well as the Munro et al. work pioneering the tiered TTC concept subsequently applied to each of its three classes on the basis of toxicity potency distributions, were pivotal advancements in the risk assessment of data-poor substances such as impurities (Cramer et al., 1978; Munro et al., 1996). The *in silico* implementation of the Cramer scheme again presents significant advancement in the accessibility of such an approach to risk assessors. As with all other models or expert judgements, these are useful but not infallible, and are of course subject to continuous evolution. Our work has, nevertheless, identified opportunities to further refine and increase confidence in their adoption.

## Supplementary Material

SI

## Figures and Tables

**Table 1:** Composition of the source collections each integrated into the unified dataset. “Defined structure” indicates substances for which a discrete chemical structure could be traced (and which were thus eligible for use *in silico*).

Data collection	Expert classification source	n. Substances classified	
	
		Total	Defined structure

FEMA	FEMA	2088	2045
IOFI	EFSA	2845	2827
	JECFA	2147	2129
Yang et al.	Publication authors	543	506

**Table 2:** Extent of overlap in the identity of substances appearing within lists compiled by respective expert bodies. Degree to which the classification of these shared compounds is found to align is expressed in terms both of raw (percentage) concordance and weighted Cohen κ statistic.

			Expert classification source		
	
			EFSA	JECFA	Yang et al.

Expert class. Source	FEMA	*n.* Substances shared	1988	1969	130
		% Class concordance	97.0	99.8	83.1
		κ_(weighted)_	0.948	0.995	0.674
	EFSA	*n.* Substances shared		2147	150
		% Class concordance		96.9	82.7
		κ_(weighted)_		0.949	0.681
	JECFA	*n.* Substances shared			135
		% Class concordance			83.7
		κ_(weighted)_			0.683

**Table 3:** Overview of concordance in Toxtree original-, Toxtree revised- and QSAR Toolbox-sourced Cramer classifications.

A		Toxtree revised			% Concordance		K *(conf. interval)*	
	Class	I	II	III	Class	Overall	Weighted	Unweighted
Toxtree original	I	1570	154	164	69.8	68.3	0.525 (0.500–0.551)	0.431 (0.406–0.455)
	II	154	40	40	5.71			
	III	208	313	612	45.8			
B		QSAR Toolbox			% Concordance		K *(conf. interval)*	
	Class	I	II	III	Class	Overall	Weighted	Unweighted
Toxtree original	I	1628	79	147	76.4	82.6	0.718 (0.695–0.741)	0.665 (0.641–0.69)
	II	148	49	29	15.4			
	III	130	13	912	74.1			
C		QSAR Toolbox			% Concordance		K *(conf. interval)*	
	Class	I	II	III	Class	Overall	Weighted	Unweighted
Toxtree revised	I	1620	70	200	74.4	71.5	0.556 (0.530–0.582)	0.474 (0.449–0.499)
	II	126	44	310	7.63			
	III	160	27	578	45.3			

**Table 4:** Overview of concordance between selected expert (i.e., EFSA and Yang et al.) and *in silico*-derived Cramer class assignments. Bar length represents sum of expert-issued judgments per class, whereas colour indicates quantity of corresponding Toxtree verdicts (Class I as green, II as yellow and III as orange).

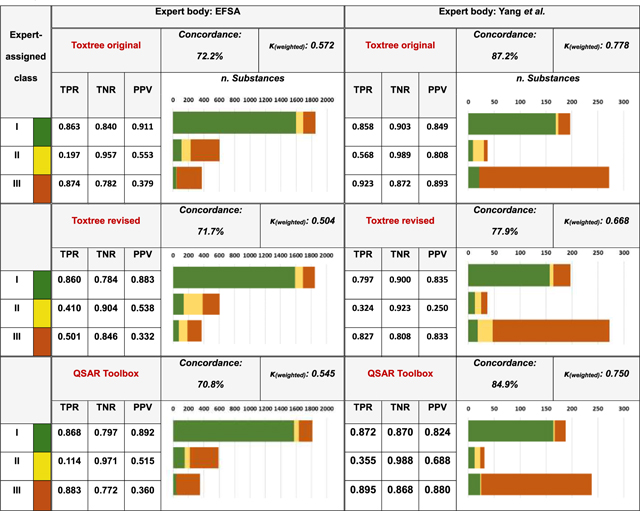

**Table 5:** Substance groups identified as leading drivers of inter-source inconsistency with respect to assignment of Cramer class. “Key rules” within each source (as appropriate) are those most central in determining divergence. Further details regarding their roles are provided within “Explanatory notes”.

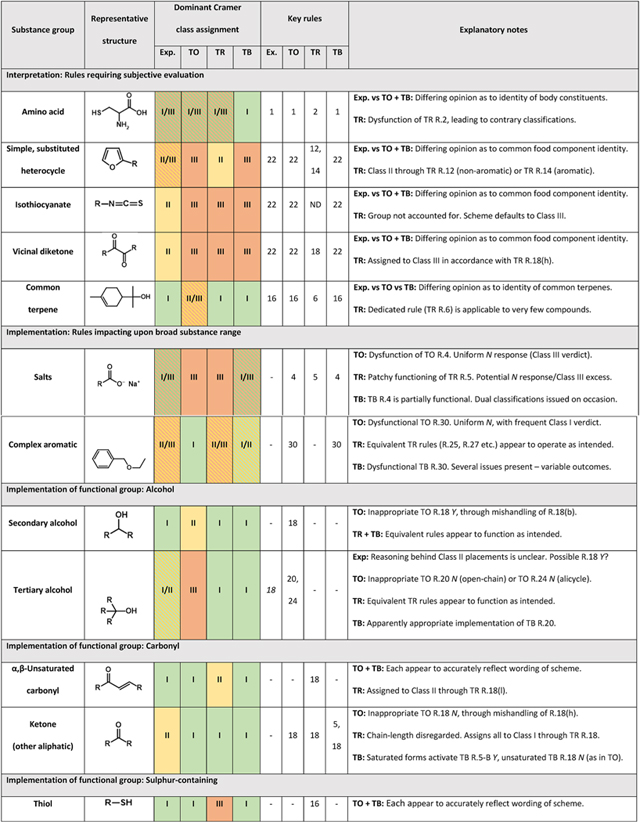	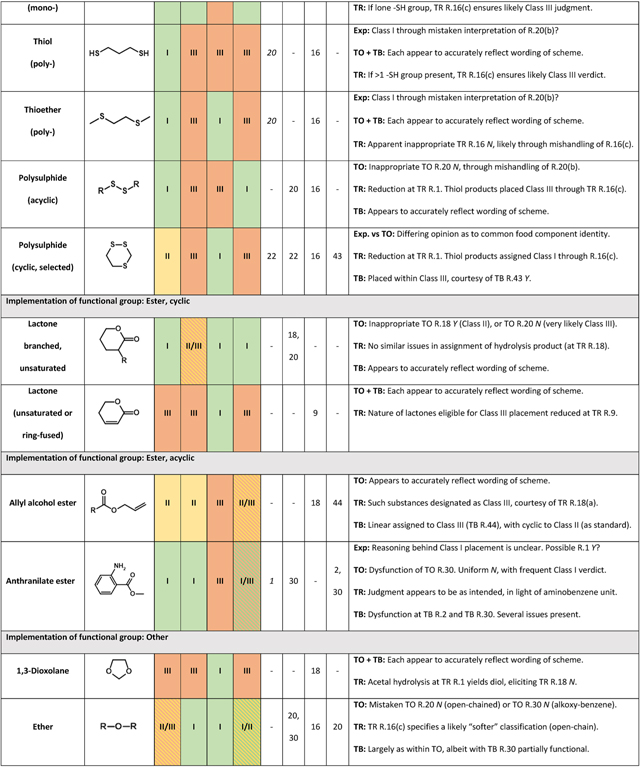	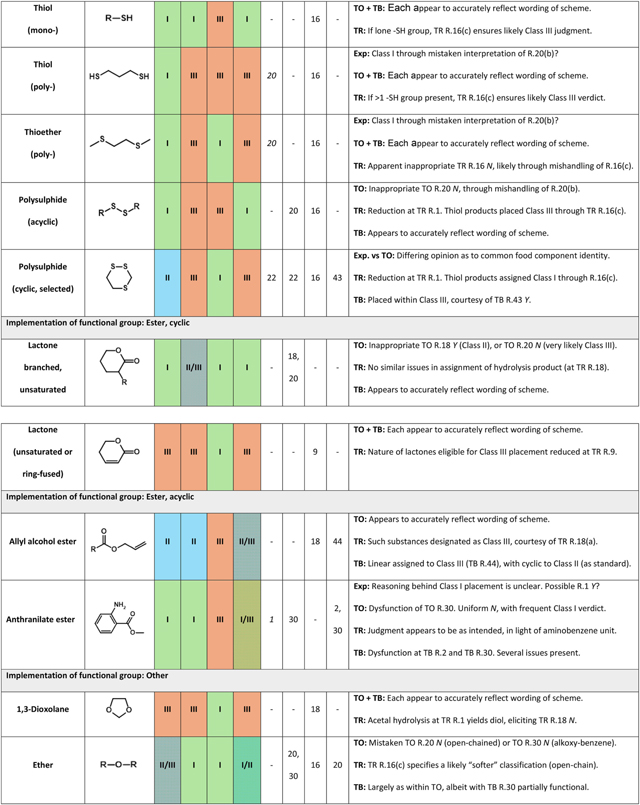

## Abbreviations

CAS: Chemical Abstract Service
DTXSID: DSSTox Substance Identifiers
ECFA: European Committee for Future Accelerators
EFSA: European Food Safety Authority
Exp: Expert Judgement
FEMA: Flavour and Extract Manufacturers Association
IOFI: International Organisation of the Flavour Industry
JECFA: Joint FAO/WHO Expert Committee on Food Additives
PPV: Positive Predictivity Value
QSAR: tool box Quantitative Structure Activity Relationship
TB: QSAR Toolbox implementation of Cramer Scheme
TNR: True Negative Rates
TO: Toxtree Implementation of Original Cramer Scheme
TPR: True Positive Rates
TR: Toxtree Implementation of Revised Cramer Scheme
TTC: Threshold of Toxicological Concern
